# The effect of chair massage on muscular discomfort in cardiac sonographers: a pilot study

**DOI:** 10.1186/1472-6882-10-50

**Published:** 2010-09-16

**Authors:** Deborah J Engen, Dietlind L Wahner-Roedler, Anita M Nadolny, Colleen M Persinger, Jae K Oh, Peter C Spittell, Laura L Loehrer, Stephen S Cha, Brent A Bauer

**Affiliations:** 1 Department of Physical Medicine and Rehabilitation, Mayo Clinic, Rochester, Minnesota, USA; 2 Division of General Internal Medicine, Mayo Clinic, Rochester, Minnesota, USA; 3 Division of Cardiovascular Diseases, Mayo Clinic, Rochester, Minnesota, USA; 4 Division of Biomedical Statistics and Informatics, Mayo Clinic, Rochester, Minnesota, USA

## Abstract

**Background:**

Cardiac sonographers frequently have work-related muscular discomfort. We aimed to assess the feasibility of having sonographers receive massages during working hours in an area adjacent to an echocardiography laboratory and to assess relief of discomfort with use of the massages with or without stretching exercises.

**Methods:**

A group of 45 full-time sonographers was randomly assigned to receive weekly 30-minute massage sessions, massages plus stretching exercises to be performed twice a day, or no intervention. Outcome measures were scores of the *Quick*DASH instrument and its associated work module at baseline and at 10 weeks of intervention. Data were analyzed with standard descriptive statistics and the separation test for early-phase comparative trials.

**Results:**

Forty-four participants completed the study: 15 in the control group, 14 in the massage group, and 15 in the massage plus stretches group. Some improvement was seen in work-related discomfort by the *Quick*DASH scores and work module scores in the 2 intervention groups. The separation test showed separation in favor of the 2 interventions.

**Conclusion:**

On the basis of the results of this pilot study, larger trials are warranted to evaluate the effect of massages with or without stretching on work-related discomfort in cardiac sonographers.

**Trial Registration:**

NCT00975026 ClinicalTrials.gov

## Background

Ultrasonography is an essential health care diagnostic service. However, the activities performed by ultrasound technicians often result in work-related injuries, especially in those with heavy workloads and those who have been in the profession for many years [1-3].

Numerous studies have documented musculoskeletal injuries and symptoms among sonographers. Whereas the point prevalence for neck and upper limb pain in the general population is 13% to 22%, for sonographers it is between 63% and 91% [2]. These problems are associated with a considerable level of disability: 80% of sonographers seek treatment for musculoskeletal injuries [4], 46% use physiotherapy or medication to control pain [5], 16.7% miss work as a result of symptoms, 9.4% decrease their hours, 14.6% decrease their regular duties, 21.2% use sick leave, and 11.75% use vacation days [6]. In addition, according to the Sonography Benchmark Survey, more than 80% of sonographers work while in pain, and 20% of these professionals eventually have a career-ending injury [7].

Ultrasound examinations require a particular type of muscular effort on the part of the sonographer. Tiny muscular tears that are the result of repetitive manipulations of the transducer, without adequate rest between examinations, can progress to more extensive muscular damage. Industry standards have been introduced to address this problem [8,9]. Because of intense work schedules, however, it is often difficult for full-time workers to participate in such programs.

Massage therapy has been shown to affect both the structure and function of the musculoskeletal system by promoting a relaxation response, decreasing muscle tension, and decreasing tonic muscle contractions [10]. Introducing massage into the workplace might have a beneficial effect on common symptoms experienced by sonographers. We therefore performed a pilot study of massage therapy in the workplace, with or without stretching exercises, for cardiac sonographers. By using separation tests as described by Aickin [11,12], we aimed to determine whether a larger trial evaluating these measures should be recommended. We hypothesized that massage could be effectively delivered in the work environment and that it would have positive effects on muscular discomfort in sonographers.

## Methods

### Subjects

This study was approved by the Mayo Clinic Institutional Review Board. There are 2 cardiac echocardiography laboratories at Mayo Clinic, Rochester, Minnesota, which employ 90 cardiac sonographers. Invitations to participate in the study were sent to all cardiac sonographers by e-mail, and the details about the trial were posted on the Echocardiography Laboratory Web site.

### Study Design

**Figure 1:**
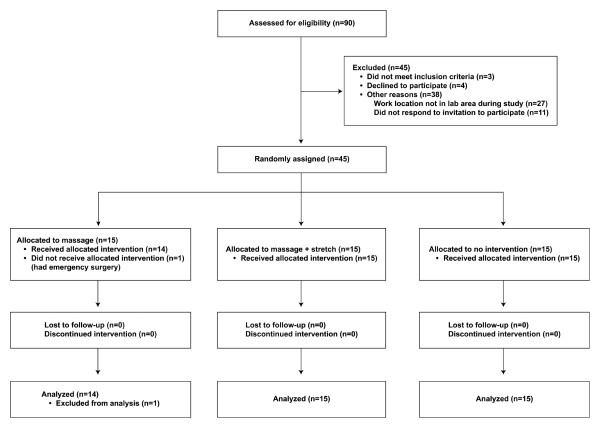
**Flowchart of Patient Recruitment and Retention**.

From October 2 through December 23, 2008, we conducted a 10-week randomized controlled early-phase trial. Those who responded to the invitation to participate were randomly assigned to 1 of 3 groups: massage therapy alone ("massage group"), massage therapy plus stretching exercises ("massage+stretch group"), or no intervention (control group) (Figure 1). A simple randomization list for 3 treatment arms was generated before the study. Treatments were balanced within each block of 15 participants.

For the massage group, massage therapy was provided as a chair massage by 1 of 3 Certified Massage Therapists in a 30-minute scheduled session once a week. For the massage+stretch group, subjects had a 30-minute chair massage weekly, as in the massage group; they also received instruction in stretching exercises and were asked to perform the stretches twice daily for 20 minutes at a time during work hours. The control group (no intervention) was given gift certificates for 5 20-minute massages to be provided after completion of the study. Informed consent was obtained from all participants.

The sonographers in the 2 intervention groups were required to schedule their massage sessions during working hours using their scheduled break times or cross-coverage; 1 of 3 Certified Massage Therapists was available for 3 hours a day in an area adjacent to the echocardiography laboratory. The study coordinator kept records of compliance with the protocol.

### Instruments Used

All participants completed the *Quick*DASH--a shortened version of the DASH (Disabilities of the Arm, Shoulder, & Hand) Outcome Measure--and its associated work module at the start and completion of the study. The *Quick*DASH Outcome Measure is a validated instrument [13-15] developed by the Institute for Work & Health and the American Academy of Orthopaedic Surgeons and supported by numerous national organizations. As described in "Scoring the *Quick*DASH" [16], the *Quick*DASH is scored in two components: the disability/symptom section (11 items, scored 1-5) and the optional high performance sport/music or work modules (4 items, scored 1-5) (reproduced here with permission).

Disability/symptom score. At least 10 of the 11 items must be completed for a score to be calculated. The assigned values for all completed responses are simply summed and averaged, producing a score out of five. This value is then transformed to a score out of 100 by subtracting one and multiplying by 25. This transformation is done to make the score easier to compare to other measures scaled on a 0-100 scale. A higher score indicates greater disability.

There are two optional modules, each consisting of four items. The optional modules are intended for athletes, performing artists and other groups of workers whose jobs require high levels of physical performance. We used the work module in this study. The same procedure described for the disability/symptom score is followed to calculate the optional four-item module score. All four questions must be answered in order to calculate the score.

### Interventions Used

#### Chair Massage

Three Certified Massage Therapists, with experience ranging from 2.5 to 5 years, performed the massage therapy in this study. Before the start of the study, the 3 therapists worked with an Occupational Therapist/Certified Massage Therapist in gaining understanding of a cardiac sonographer's job functions, common pain, tension problem areas, and common resulting injuries. The massage therapists demonstrated consistency in individualized assessment, communication, and gaining treatment consensus with a client, and in providing muscle release and connective tissue release techniques with chair massage.

The chair massage sessions were provided in a private to semiprivate work area next to the echocardiography laboratories. Each participant remained clothed for the session. Using a massage chair, the person rests in a sitting, semikneeling position leaning forward with the torso and arms supported and the face resting in a face cradle. A disposable face cradle cover is used for each session. This position allows the therapist to use massage techniques for the scalp, neck, shoulders, arms, hands, back, and hips.

Each therapist completed a brief visual and verbal assessment with ongoing palpation throughout each session. Primary techniques used during the massage sessions were compression, cross-fiber friction, pressure point release, trigger point release, percussion, vibration, and range of motion/stretching techniques. The therapists used and adjusted techniques, pace, and pressure on the basis of each person's musculoskeletal needs and physiologic responses. Each session ended with stimulation strokes to help the participant return to work optimally alert.

#### Stretching Exercises

Twelve stretching exercises were selected (Bodyworks Program "Stretch Sheet for Echocardiographers" [17]) on the basis of the experience of physical and occupational therapists in our Department of Physical Medicine and Rehabilitation work rehabilitation section who frequently work with injured sonographers. Each stretch exercise was to be held for 30 seconds in several directions, with an average of 20 minutes to complete the 12 stretches. Instructions on the stretching program were provided and demonstrated to each participant in a session before the study. Participants were instructed to document dates and times they completed the stretches.

### Sample Size and Power

A sample size of 15 in each group was originally planned, which had 80% power to detect a pre-post effect size of 0.778 (or difference of 0.778 × SD) for pre-post testing within each group using paired *t *tests. This sample size also had 80% power to detect an effect size of 1.060 (or mean difference of 1.060 × common SD) comparing each experimental arm and the control arm using a 2-sample *t *test. All *t *tests were 2-sided with a .05 significance level.

### Statistical Analysis

Patient demographics were summarized using descriptive statistics. The mean (SD) of the total *Quick*DASH scores and the work module scores at baseline and at 10 weeks were calculated for each subject group. The difference in the mean *Quick*DASH scores and work module scores from baseline to 10 weeks was analyzed using a paired *t *test with intent-to-treat analysis. *P *< .05 was considered statistically significant.

Because this study was an early-phase trial with a small sample size, we also analyzed our data by using the separation test, as described by Aickin [11,12], to assess whether it is worthwhile to pursue further research on massage with or without stretch therapy. By use of this test, the standard deviation of the effect estimate (SDE) of the mean difference can be found. The value of Δ (1.645 × SDE) is then calculated. If the mean difference exceeds Δ/2 (in the direction favorable to the intervention), further research is recommended; if it decreases below -Δ/2 (in the direction unfavorable to the intervention), further research is not recommended. Otherwise, if the mean difference falls between these limits, not enough information is available to make a recommendation.

## Results

### Baseline Characteristics

A total of 45 cardiac sonographers (37 women, 8 men) were enrolled in this study. Median age was 33 years (range, 22-53 years). Median duration of employment as sonographer was 5 years (range, 6 months to 30 years). The 45 subjects were divided evenly among the 3 study groups; all but 1 subject, in the massage group, completed the study. Although the groups were randomly assigned, the baseline *Quick*DASH disability/symptom scores and work module scores were significantly lower (meaning less disability) in the control group than in the other 2 groups (*P *= .02). No significant differences were seen among the groups in sex, age, work days missed due to work-related pain by the end of week 10, and duration of employment as a cardiac sonographer.

### Compliance With Program

The average number of massages performed during the study period for the massage and massage+stretch groups were 9.6 and 9.8, respectively (of 10 possible). Stretching sessions in the massage+stretch group averaged 7.7 per week (of 10 requested).

### Within-Group Comparisons

Because no baseline records were available for the 1 participant who enrolled but then was unable to participate, we excluded this patient from all analyses. The *Quick*DASH disability/symptom score decreased in the intervention groups and increased in the control group from baseline to completion (Table 1). Work module scores decreased in all groups, but the difference was statistically significant only in the massage+stretch group (*P *= .008).

**Table 1:** Disability Scores Before and After Intervention

	**Group**^**a**^					
	
Score	Massage (n = 14)	***P***^**b**^	Massage + Stretch (n = 15)	***P***^**b**^	Control (n = 15)	***P***^**b**^
*Quick*DASH		.08		.06		.80
Baseline	18.18 (13.92)		11.33 (8.07)		7.36 (5.95)	
10 weeks	12.66 (16.28)		8.03 (5.82)		7.88 (10.09)	
Difference^c^	5.52 (10.83) [-15.72-19.97]		3.30 (6.24) [-8.93-11.26]		-0.52 (7.54) [-15.29-22.43]	
Work module		.06		.008		.90
Baseline	22.77 (16.74)		17.08 (13.04)		10.83 (9.87)	
10 weeks	16.07 (18.30)		7.50 (8.90)		10.42 (10.48)	
Difference^c^	6.70 (12.37) [-17.55-22.03]		9.58 (12.01) [-13.96-15.36]		0.42 (12.82) [-24.72-35.62]	

^a ^Values are mean (SD).

^b ^Paired *t *test for change in score.

^c ^Baseline-10 weeks [95% confidence interval].

### Between-Group Comparisons

The separation test using the difference in the *Quick*DASH disability/symptom and work module scores from baseline to 10 weeks showed a benefit for the massage and the massage+stretch groups over no intervention (Table 2), which indicates that further research using massage with or without stretching is reasonable. The analysis further showed separation in favor of massage alone using the *Quick*DASH scores and separation in favor of massage plus stretching using the work module scores.

**Table 2:** Separation Tests

	Analysis			
	
Comparison	**Difference in Score**^**a**^	SDE	**Δ/2**^**b**^	**Separation/In Favor Of**^**c**^
Massage vs Control				
*Quick*DASH	6.04	1.78	1.47	Yes/Massage
Work module	6.28	2.37	1.95	Yes/Massage
Massage + Stretch vs Control				
*Quick*DASH	3.82	1.29	1.06	Yes/Massage+Stretch
Work module	9.16	2.39	1.96	Yes/Massage + Stretch
Massage vs Massage + Stretch				
*Quick*DASH	2.22	1.61	1.32	Yes/Massage
Work module	-2.88	2.24	1.84	Yes/Massage+Stretch

Abbreviation: SDE, standard deviation of the effect estimate.

^a ^Difference between the 2 interventions in the mean change in score ("Difference" in Table 1.)

^b ^Δ = 1.645 × SDE.

^c ^If the difference in score is greater than Δ/2 (in the direction favorable to the intervention), further research is recommended.

## Discussion

Chair massage, using a padded, ergonomically designed, portable chair, has become increasingly popular in work environments because of its adaptability and method of delivery [10,18-21]. This study demonstrated the feasibility of incorporating chair massage into the workflow of a busy echocardiography laboratory. Compliance with the intervention was high. In addition, *Quick*DASH disability/symptom and work module scores generally improved with the interventions. Analysis using the separation test suggests that these preliminary findings are sufficient to warrant a larger-scale trial. These results are particularly important given the challenges faced by sonographers and the relative paucity of effective interventions.

Exercises and stretching programs have been developed specifically for sonographers to help strengthen the torso and upper extremities [16]. Recently, wellness programs have become popular. However, considering the workload of full-time cardiac sonographers, in addition to obligations outside the workplace, it may be difficult for them to find the time to participate in these programs. We therefore wanted to provide sonographers with an "in-lab" opportunity for massage and stretching during their working hours. The technicians had to work out their own schedule with the massage therapist.

The chair massage sessions and stretches were designed to focus on the musculoskeletal imbalance areas in cardiac sonographers that often lead to injury. We chose chair massage over table massage for feasibility reasons. A massage chair is easy to set up, does not need much space, and can be provided in semiprivate areas. Chair massage therapy addresses the scalp, neck, shoulders, arms, hands, back, and hips, which are the primary musculoskeletal imbalance areas in cardiac sonographers. The cardiac sonographer is usually sitting and reaching forward or to the side, and the musculoskeletal imbalances show up primarily superior to the hips, which leads to possible shortening of muscles. This in turn can result in joint compressions and imbalance of normal joint motions, leading to joint wear, nerve impingement, muscle fatigue, and weakness.

This early-phase study demonstrated the feasibility of providing chair massage; most sonographers were able to schedule and receive once-weekly massage therapy sessions, and some could perform stretching exercises twice daily. However, the study has several limitations. First, the study was small and, hence, not powered to allow definitive statements about the role of massage therapy. The study was also of short duration. Thus, future studies are needed to explore the optimal frequency and duration of chair massage. For example, it is possible that offering more frequent massages initially (eg, 3 times a week) could lead to an initially more notable response. Determining optimal maintenance dosing of massage (assuming an initial positive response is achieved) would similarly be an important area for exploration. Longer-term studies will also be needed to assess the benefit and cost-effectiveness of massage therapy with or without stretching exercises provided at work. The current findings support the need for developing such studies that could lead to an important intervention for promoting health and wellness for sonographers.

## Conclusion

Sonographers were able to schedule and undergo weekly 30-minute massage sessions and perform stretches twice daily during working hours using an "in-lab" facility. Some improvement of work-related discomfort was seen in the intervention group, as measured by *Quick*DASH Outcomes Measure scores and work module scores. A larger study is needed to arrive at a definite conclusion regarding the usefulness of massage therapy with or without stretching exercises offered during working hours for cardiac sonographers.

## Abbreviations

DASH: Disabilities of the Arm, Shoulder, & Hand; SDE: standard deviation of the effect estimate.

## Competing interests

The authors declare that they have no competing interests.

## Authors' contributions

D.J.E. contributed to the conception and design, interpretation of data, and drafting of the manuscript. D.L.W.-R. made substantial contribution to the conception and design, interpretation of data, and drafting of the manuscript. A.M.N. participated in the design and coordination of the study and reviewed the manuscript. C.M.P. participated in the design and coordination of the study and reviewed the manuscript. J.K.O. helped in the design of the study and manuscript review. P.C.S. participated in the design of the study and manuscript review. L.L.L. participated in the design and coordination of the study and helped to draft the manuscript. S.S.C. participated in the design of the study and the analysis and interpretation of data. B.A.B. participated in the design and coordination of the study and helped to draft the manuscript. All authors read and approved the final manuscript.

## Pre-publication history

The pre-publication history for this paper can be accessed here:

http://www.biomedcentral.com/1472-6882/10/50/prepub
